# Editorial: Role of Phytochemicals and Structural Analogs in Cancer Chemoprevention and Therapeutics

**DOI:** 10.3389/fphar.2022.865619

**Published:** 2022-03-16

**Authors:** Jamal M. Arif, Raghuram Kandimalla, Farrukh Aqil

**Affiliations:** ^1^ Department of Biochemistry, College of Medicine, Shaqra University, Shaqra, Saudi Arabia; ^2^ UofL Health—Brown Cancer Center, University of Louisville, Louisville, KY, United States; ^3^ Department of Medicine, University of Louisville, Louisville, KY, United States

**Keywords:** phytochemicals, cancer, chemoprevention, structural analogous, chemotherapy—oncology

Cancer is a multifactorial disease and a leading cause of deaths worldwide. An estimated 1.89 million new cancer cases and about 600,000 cancer related deaths were reported only in United Stated in the year 2021 (Siegel et al., 2021). A systematic and complicated mechanism is involved in the transformation of a normal cell into cancerous form. In human body, most cell functions are controlled by genes through cell growth, signal transduction, protein transcription, cell cycle, apoptosis and DNA repair. While proto-oncogenes are essential for the normal functioning of cells, mutations covert them into oncogenes and lead to uncontrolled cell growth. There are about 40 different proto-oncogenes discovered to date including RAS, HER2, Myc and cyclins. The treatment options for cancers involve surgery followed by chemotherapy and/or radiation. More than 100 chemotherapeutic drugs are in use to treat cancer of different types. However, the efficacy of chemotherapy of cancer is limited by the development of drug resistance and metastatic disease.

Lately, plant bioactives are gaining increased attention for their therapeutic activity against many cancers (nearly 2000 publications per year) as they possess versatile biological properties and target multiple pathways in cancer. Plant-derived compounds have historically led to some of our most useful cancer drugs (e.g., paclitaxel, vincristine, etc.). These chemopreventives can block initiation, reverse promotion and/or halt the progression of precancerous cells into malignant cells. Since advanced, recurrent and metastatic tumors are practically lethal and cannot be cured by any therapy, cancer chemoprevention of earlier lesions should be the definitive goal if we are to eradicate this deadly disease. To fulfil the unmet needs of cancer treatments, a scrupulous repurposing of plant-based phytochemicals such as chemical modification of the pharmacophores, development of target-based delivery strategies including nano formulations, and their uses in adjuvant settings need to be considered. In this regard, we launched the current special issue to highlight the current development in the anticancer drug research.

Most of the chemotherapy treatment failures occur due to drug resistance subsequently limiting its use and available treatment options. Several factors like tumor microenvironment, size, heterogeneity, involvement of immune system and undruggable genes necessitate in evoking the drug resistance (Vasan et al., 2019). For instance, over expression of myeloid cell leukemia 1 (Mcl-1) leads to resistance towards drugs used to treat multiple myeloma. Al-Odat et al. discuss the role of Mcl-1 inhibition in the management of multiple myeloma. They further highlight that the most prominent BH3 mimetic and semi-BH3 mimetic selectively inhibit Mcl-1 and could be used by itself or in combination with existing therapies for the management of multiple myeloma. In another article, Cui et al. showed that the tubulin Inhibitor VERU-111 in combination with vemurafenib, an FDA-approved BRAF inhibitor, provides an effective treatment for vemurafenib-resistant A375 melanoma.

Besides repurposing the existing drugs, discovery of novel drug molecules is the most explored alternate option to overcome chemotherapy related drug constraints in cancer therapy. In this context, new plant bioactives fill the void of limited chemotherapy options. Uterine leiomyosarcoma is a rare gynecological malignancy with no standard approved drug at present. Wet et al. successfully demonstrated using *in vitro* and *in vivo* assays that curdione isolated from *Curcuma zedoary* suppressed uterine leiomyosarcoma cell proliferation by inducing G2/M phase arrest, apoptosis, and autophagy via targeting IDO1. In the similar line, Macleayins A and phenethyl isothiocyanate were shown to inhibit the growth of cervical cancer cells (Shoaib et al.; Sai et al.).

In a systematic review and meta-analysis of randomized clinical trials, Peng et al. concluded that ginsenoside Rg 3 isolated from Korean *Panax ginseng* in combination with first line chemotherapeutic drugs demonstrate enhanced efficacy towards advanced non-small cell lung cancer (NSCLC) patients. Ginsenoside Rg 3 also reduced the chemotherapy induced toxicity. Similarly, phyto-chemicals from banana also showed great potential for future development of drugs for cancer prevention and therapy (Mondal et al.).

Despite potential anticancer abilities, many novel anticancer drugs suffer from poor bioavailability to achieve therapeutic drug level. The review by Wagh et al., provides an up-to-date summary on the effect of celastrol and its nano formulations in cancer prevention. This review discusses a multitude of celastrol nanoformulations that have been developed and tested for various therapeutic applications. Outside the scope of this review, exosomal formulations using milk derived exosomes have been demonstrated against lung cancer using *in vitro* and *in vivo* models (Aqil et al., 2016). In another review, Aggrawal et al. highlights phytochemical-related research on human papilloma virus (HPV)-induced head and neck cancer (HNC) and concluded that the therapies to distinguish HPV-positive HNC from HPV-negative HNC could help to achieve better outcome. This HPV-associated HNC can be treated with well-established phytochemicals targeting the HPV-mediated carcinogenic mechanisms.

Plant polyphenols are known for their pharmacological activities and therapeutic benefits. A study conducted by Albini et al. demonstrate that the polyphenol-rich extract of olive mill waste water enhances the anticancer potentiality of chemotherapeutic drugs cisplatin and 5-fluorouracil while mitigating their dose related cardiotoxicity. In another study, Oxyresveratrol was shown to have the ability to alter the cancer-causing genes and subsequently inducing apoptosis and cell cycle arrest in breast cancer MCF-7 cells (Radapong et al.).

Growing body of evidence suggests that approaches like chemical synthesis, analogue modification and development of nano formulations might help to achieve better anticancer therapies. Otaibi et al. shed the light on this aspect in their study and show chemically synthesized biologically active α-amino amide analogs alone and in combination with T cells exhibit pronounced anticancer activity against leukemia cancer cell lines (HL-60 and K562). In another study, Rampogu et al. used chemically modified butein derivatives to inhibit breast cancer progression through modulation of PI3K/AKT signaling pathway and other molecular markers.

In summary, this research topic comprises eight informative research and five authoritative review articles written by expert scientists and expansively discusses various aspects of cancer prevention and control. This special issue highlights strategies for cancer prevention and therapy by plant therapeutics alone and in combination with already in use therapeutics drugs, repurposed molecules for other diseases, and various nanotechnology-based techniques to enhance efficacy, reduce dose related toxicity and to better manage resistant and metastatic cancers. While articles in this special issue show *in vitro* and *in vivo* data for cancer prevention, the review articles shed light on preclinical and clinical studies in cancer prevention and therapy.
